# Assessment of Hydrochar and Porous Carbon from *Tectona Grandis* Seeds for Removal of Acridine Dyes

**DOI:** 10.3390/molecules30193989

**Published:** 2025-10-04

**Authors:** Shubham Chaudhary, Monika Chaudhary, Sarita Kushwaha, Vaishali Tyagi, Shivangi Chaubey, Isabel Pestana da Paixão Cansado, Evgeny Galunin, Suhas

**Affiliations:** 1 Department of Chemistry, Gurukula Kangri (Deemed to be University), Haridwar 249404, India; shubhamchaudhary89@yahoo.com; 2 Department of Chemistry, Motherhood University, Roorkee 247661, India; saritakushwaha31@gmail.com (S.K.); tyagivaish1@gmail.com (V.T.); shivangichaubey34@gmail.com (S.C.); 3 Department of Chemistry, Hariom Saraswati P.G. College, Dhanauri, Haridwar 247667, India; monikachoudry@gmail.com; 4 MED—Mediterranean Institute for Agriculture, Environment and Development & Change—Global Change and Sustainability Institute, Universidade de Évora, Pólo da Mitra, Apartado 94, 7006-554 Évora, Portugal; 5 Department of Chemistry and Biochemistry, School of Science and Technology, University of Évora, Rua Romão Ramalho, nº 59, 7000-671 Évora, Portugal; 6 Department of Organic and Ecological Chemistry, School of Natural Sciences, University of Tyumen (UTMN), 6 Volodarsky Str., 625003 Tyumen, Russia; evgeny.galunin@gmail.com

**Keywords:** acridine dyes, activated carbon, adsorption, biomass, hydrochar, *Tectona grandis* seeds

## Abstract

This study explores the use of lignocellulosic *Tectona grandis* seeds (TGs), hydrochar (HC-230-4), and activated carbon (AC-850-5) produced via hydrothermal carbonization and followed by CO_2_ activation for removing acridine yellow G (AYG) and acridine orange 14 (ABO) from water. HC-230-4 showed a rich presence of surface functional groups and irregular morphology with some sphere-like structures. In contrast, AC-850-5 exhibited a much higher surface area (729.7 m^2^/g), though with fewer surface functional groups than HC-230-4. The batch method was used to study the effects of contact time, pH, dye concentration, and temperature. Among the materials, AC-850-5 showed the highest adsorption capacity of 198 mg/g for AYG and 171 mg/g for ABO at 25 °C, around 12% higher than commercial activated carbon. The adsorption process was spontaneous and endothermic, fitting well to the Langmuir isotherm model, suggesting monolayer coverage. The adsorption kinetics followed the pseudo-second-order model, indicating that the rate depends on the surface site availability. Intraparticle diffusion analysis further confirmed a multi-step adsorption process. These findings show the strong potential of TG-derived activated carbon as an effective and sustainable material for removing acridine dyes from polluted water.

## 1. Introduction

Water, the foundation of life, is facing an unprecedented crisis. The rapid growth of industrial activities, agricultural practices, and urbanization has led to a significant increase in water pollution, threatening the very sustainability of our planet. In this context, one significant contributor to water pollution is the extensive use of synthetic dyes in industries like textiles, leather, and paper. These colored substances brighten our textiles and enhance our products but they pose an unimaginable threat to the environment. When discharged into waterways without proper treatment, they can disrupt the delicate balance of aquatic ecosystems, harming both human health and aquatic life [1,2].

Due to their aromatic structure, synthetic dyes exhibit resistance to heat and oxidizing agents, presenting also a potential to induce cancerous and mutagenic diseases in humans [3,4,5]. The demand for synthetic dyes has substantially increased, in recent decades, with approximately 7 × 10^5^ metric tons of dyes. It includes around 1 × 10^6^ different types (cationic, anionic, disperse, and vat dyes), being manufactured annually for commercial purposes [6,7,8]. Among these, cationic dyes have garnered attention due to their excellent solubility, persistence in water, and recalcitrant effect [8]. Additionally, they have a very high tinctorial value, such as a concentration as low as < 1 ppm which is sufficient to bring color to the solution [9]. Cationic dyes belong to various chemical classes in which thiazine, triarylmethane, oxazine, cyanine, hemicyanine, diazahemicyanine, and acridine are the principal ones [4,10].

Acridine dyes are widely used for various applications, including dyeing silk, synthetic fibers and leather, high fluorescence intensity in luminescent analysis, as well as for printing purposes [11]. Acridine orange (AO) dyes are referred to as being an organic compound that serves as a nucleic acid selective fluorescent dye, such as it presents the cationic properties useful for cell cycle determination. They are mostly used in medicine and biology for staining solutions [12]. Acridine dyes have been found to possess mutagenic potential, owing to their ability to damage DNA. Exposure to these dyes can result in adverse health effects, including eye and skin irritation, as well as respiratory problems upon ingestion or inhalation [13]. Hence, they must be eliminated from wastewaters before discharging them into the environment.

A number of techniques including adsorption, biosorption, coagulation–flocculation, biological methods, ozonation, membrane separation, precipitation, and electro-chemical have been applied for the removal of these dyes from the water systems [14,15,16,17,18,19,20,21]. Among these, adsorption, mainly due to its simplicity of operation, economic viability, high removal potential, and the diversity of available adsorbents, is widely preferred for dye removal applications [22]. In the adsorption process, activated carbons have proven to be efficient and versatile materials as adsorbents for the elimination of dyes [23,24]. However, the high manufacturing cost of activated carbons (ACs) has prompted researchers to search for cost-effective alternatives [25].

Biomass, such as lignocellulosics, municipal, and sewage wastes, are abundant and renewable sources that can be converted into carbon-rich products through thermochemical methods. Various approaches have been explored in the literature to transform biomass into carbon-rich materials, including pyrolysis, torrefication, gasification, and hydrothermal carbonization [24,26,27,28]. Among these techniques, hydrothermal carbonization has emerged as a promising method for converting biomass into carbon-rich products under relatively mild conditions. The hydrochars obtained were intensively utilized for soil remediation, energy storage, fuel applications, adsorption of pollutants, and as a raw material to produce ACs. Although hydrochars have been widely used in the removal of dyes, their limited porosity is a barrier to their high performance in adsorption processes. Therefore, the activation of these materials, with the high proportion of carbon, can be an effective approach for the production of excellent adsorbents, such as activated carbon, which exhibits a high porous volume and superficial area.

In this work, we aim to evaluate and compare the adsorption capacity of raw *Tectona grandis* seeds (TGs), hydrochar (HC-230-4) and activated carbon (AC-850-5) prepared via hydrothermal carbonization followed by CO_2_ activation for the removal of acridine yellow G (AYG) and acridine basic orange 14 (ABO) dyes from an aqueous solution. Batch experiments were conducted to assess the effect of pH, contact time, dye concentration, and temperature. The adsorption behavior was further analyzed using isotherm, kinetic, and thermodynamic models. Commercial activated carbon (CAC) was also included for benchmarking. To the best of our knowledge, no study has yet provided a comparative evaluation of acridine dye removal using hydrochar and activated carbon derived from *Tectona grandis* seeds. This work offers new insights into the potential of this biomass as a sustainable and efficient adsorbent source.

## 2. Results

The adsorbents previously prepared (TGs, HC-230-4, AC-850-5) were tested for the removal of two acridine dyes from the aqueous phase. The influence of a variety of parameters on the performance of HC-230-4 and AC-850-4 for the adsorption of both dyes was evaluated, which are detailed in the following sections. Commercial activated carbon (CAC) has also been tested for the removal of acridine dye for comparison purposes.

### 2.1. Effect of Contact Time and Initial Concentration of Acridine Dyes

**Figure 1:**
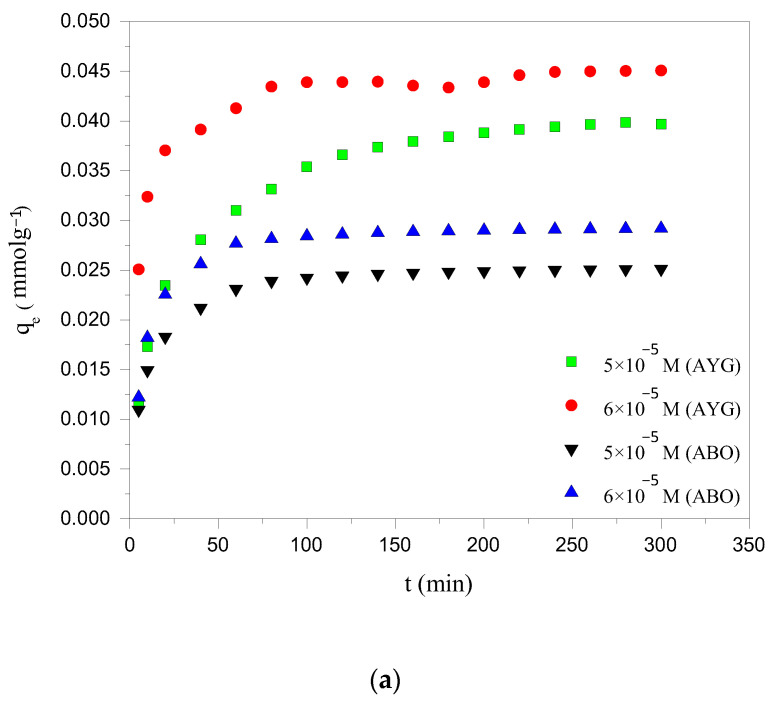

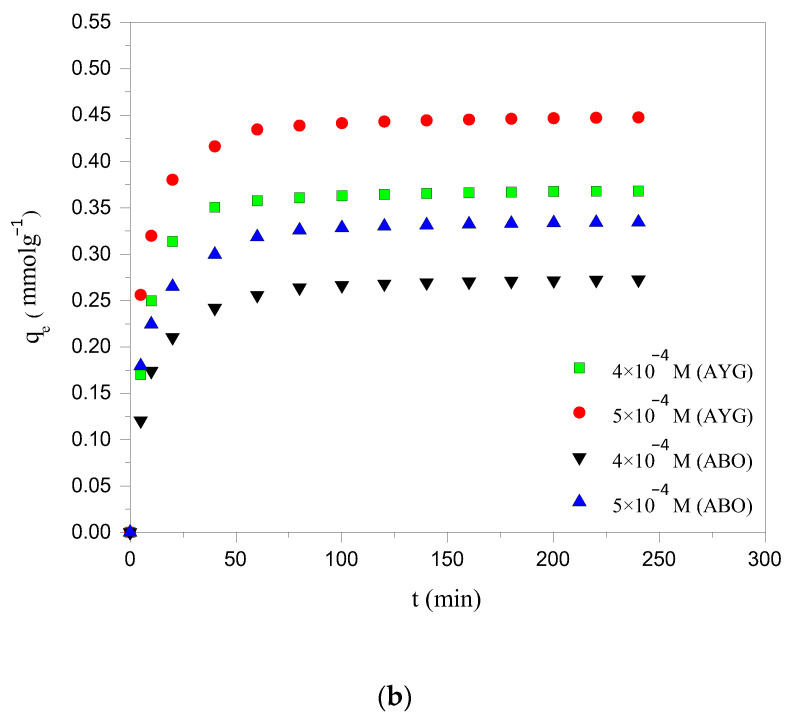
Effect of contact time and initial concentration on the adsorption of AYG and ABO onto (**a**) HC-230-4 (C_i_: 5 × 10^−5^; 6 × 10^−5^ mol dm^−3^; T: 25 °C) and (**b**) AC-850-5 (C_i_: 4 × 10^−4^; 5 × 10^−4^ mol dm^−3^; T: 25 °C).

The equilibration time is one of the most crucial parameters for achieving cost effective wastewater treatment efficiency. The influence of contact time on the removal of modeled dyes, namely AYG and ABO, by the prepared hydrochar (HC-230-4) and activated carbon (AC-850-5) was investigated, up to 300 min, at 25 °C. It is apparent from Figure 1a,b that the amount of dyes removed from the aqueous phase increases with contact time, reaching equilibrium at 240 min for HC-230-4 and 180 min for AC-850-5. Initially, AYG and ABO were rapidly adsorbed due to the abundance of free active sites on the adsorbent surfaces. As time progressed, the rate of adsorption decreased as these sites became occupied, eventually reaching saturation [29].

The effect of the initial dye concentration on adsorption was studied using two concentrations: 5 × 10^−5^ and 6 × 10^−5^ mol dm^−3^ for HC-230-4, and 4 × 10^−4^ and 5 × 10^−4^ mol dm^−3^ for AC-850-5, as shown in Figure 1a,b. The results showed that the adsorption capacity increased with the increasing dye concentration for both adsorbents. This is because, at higher concentrations, the larger driving force promotes more rapid dye transfer from the solution to the adsorbent surface, until the available active sites become saturated. Moreover, the increase in the initial dye concentration increased the adsorbent loading potential [30]. This may be attributed to an enhancement in the driving force for adsorption due to the increased concentration gradient between the liquid and solid phases, which overcomes the resistance to mass transfer [31].

### 2.2. Influence of pH of Solution on Dye Adsorption

**Figure 2:**
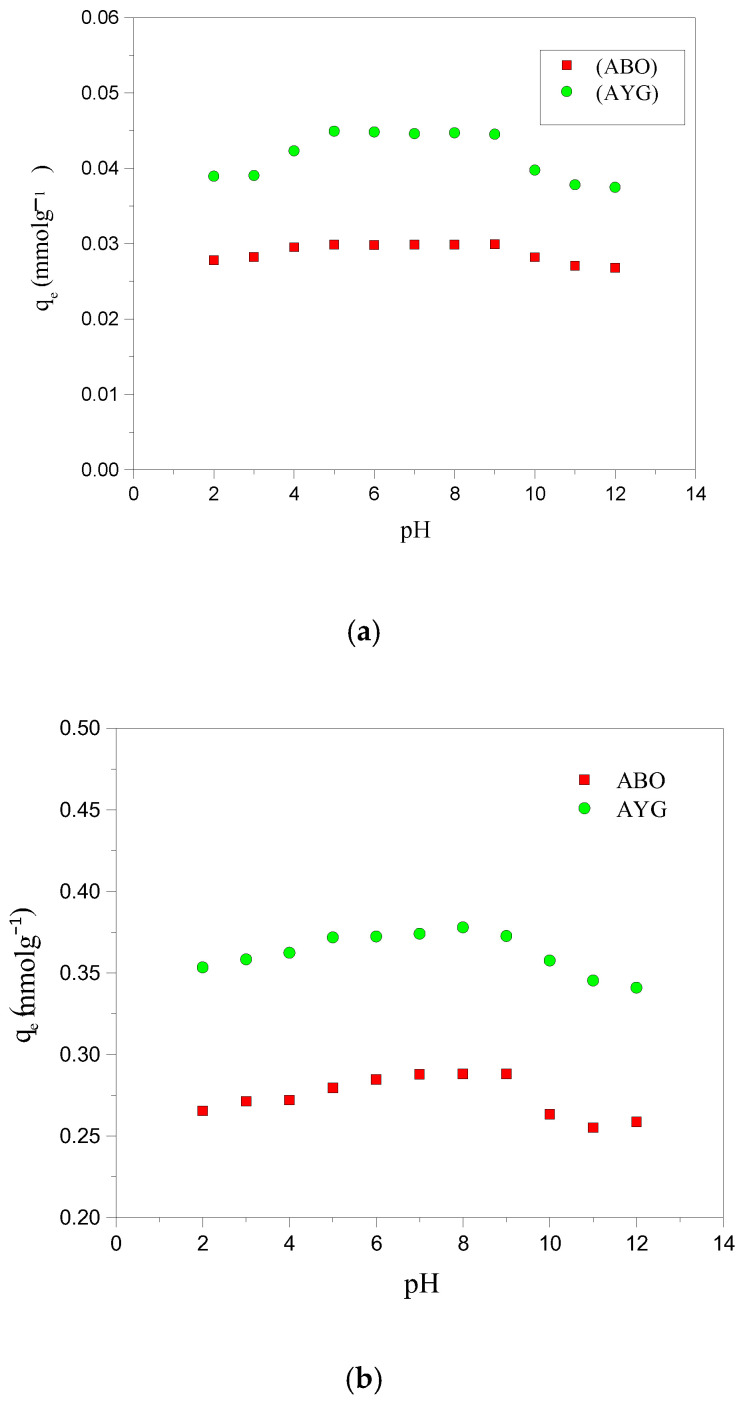
Effect of pH on the adsorption of AYG and ABO onto (**a**) HC-230-4 (Ci: 6 × 10^−5^ mol dm^−3^; T: 25 °C) and (**b**) AC-850-5 (Ci: 4 × 10^−4^ mol dm^−3^, at 25 °C).

The pH of the adsorbate solution is also a crucial factor influencing the adsorption progress. A thorough investigation of the adsorption of AYG and ABO on HC-230-4 and AC-850-5, at pH values ranging from 2 to 12, as shown in Figure 2a,b, was conducted in order to understand the impact of pH on these adsorption processes. The initial pH of the modeled dyes solutions, before pH adjustment, was 8.3 for AYG and 6.4 for ABO.

As shown in Figure 2a,b, maximum adsorption for both dyes occurred within the pH range of 5–9 for both adsorbents. At lower pH values (pH < 4.3 for HC-230-4 (hydrochar) and < 6.2 for AC-850-5), the reduced adsorption capacity can be primarily attributed to competition between H^+^ ions and dye cations for active sites, along with the proto-nation of surface functional groups that suppress dye binding. Electrostatic repulsion may also play a role at a very acidic pH (below 4.3 for HC-230-4), but from a pH of 4.3 and above, the surface of HC-230-4 becomes negatively charged, enabling electrostatic attraction with cationic dye species.

Moreover, as inferred from the FTIR analysis (Table 1), the surface of HC-230-4 is rich in oxygenated functional groups, such as hydroxyl and carboxyl groups, which contribute to dye adsorption through multiple interactions including hydrogen bonding, π–π stacking, and electrostatic attraction. For both dyes, adsorption by HC-230-4 decreased above pH 9. This decline could be attributed to multiple factors, including the potential for dye molecule aggregation or dimerization at higher pH, which reduces the number of free dye monomers available for adsorption [32,33]. At an alkaline pH, the increased concentration of OH^−^ ions may compete with dye cations for active sites or lead to electrostatic screening effects, which weaken the attractive forces between the adsorbent surface and the dye molecules, resulting in reduced adsorption.

For AYG, the adsorption trend closely follows the expected pH-dependent behavior, with an increased uptake above pH 4.3 due to electrostatic attraction and a minor decline at a higher pH likely caused by OH^−^ competition and dye aggregation. In contrast, ABO adsorption onto HC-230-4 exhibits a relatively constant profile across the pH range from 5 to 9, resembling a plateau. This behavior could result from the early saturation of available adsorption sites or potential dye aggregation and dimerization at higher pH, which reduces the availability of free monomeric ABO molecules for adsorption. Additionally, kinetic limitations or diffusional constraints within the porous structure of HC-230-4 may also contribute, particularly for larger cationic dyes like ABO. These factors combined may explain the relatively constant adsorption values across the pH range.

The AC-850-5 surface becomes negatively charged at pH values above pH 6.2 and this shift in surface charge leads to dominant electrostatic interactions with the positively charged dyes [13]. Above pH 6.2, these attractive interactions become the primary driving force for adsorption, leading to an enhanced dye uptake. However, the maximum adsorption capacity for both adsorbents did not show significant variations in terms of the solutions’ pH. Hence, the subsequent adsorption studies were performed at an original pH of both dye solutions (pH = 8.3 for AYG and pH = 6.4 for ABO).

### 2.3. Dyes’ Adsorption Isotherms

Adsorption isotherms provided detailed insights into the mechanisms relating the surface properties and affinity of an adsorbent for a specific adsorbate as revealed through the analysis of the experimental data [20,36,37]. Out of the various applied and discussed models, Langmuir, Freundlinch, Temkin, and D-R isotherm models are extensively utilized to analyze and describe the experimental data. The mathematical expression and the related parameters are presented in Table 2. The joint use of these models allows for a more accurate representation and comprehension of the data, especially when dealing with complex adsorption processes.

The Langmuir isotherm equation, which is an extensively used model [38], assumes that the adsorption occurs in a monolayer on the adsorbent’s surface, and that the adsorption energy remains alike for all adsorbate molecules. The model allows for the calculation of the maximum adsorption capacity and the adsorption energy, which can be useful in terms of understanding the adsorption behavior of the material. Utilizing Langmuir parameters, a dimensionless separation factor (R_L_), which demonstrates whether the adsorption process is favorable (0 < R_L_ < 1), unfavorable (R_L_ > 1), irreversible (R_L_ = 0), or linear (R_L_ = 1) [39], can be obtained using the following expression [40]:

(1) $$R_{L}\;=\frac{1}{1+b\;Co}$$

The Freundlich model [41], which is another extensively used model, is known for providing information about the adsorption behavior for the materials with a notable degree of surface heterogeneity and the adsorption energy varies with the availability of surface site. The Freundlich model allows for the calculation of the constant related to the maximum adsorption capacity (K_F_ and heterogeneity factor (n), as shown in Table 2. The heterogeneity factor ‘n’ gives details of whether the process is physical or chemical. Values of n between 1 and 10 demonstrates the adsorbent’s compatibility with the adsorption process [42].

The Temkin isotherm model [43] is often used to account for indirect interactions between adsorbent and adsorbate molecules. It assumes that the adsorption heat decreases linearly with the surface coverage, which is typically valid for the intermediate concentration range. In this study, the Temkin isotherm was applied in its empirical linearized form (Table 2). Although B_T_ is sometimes expressed thermodynamically as B_T_ = RT/b_T,_ this formulation can lead to dimensional inconsistencies when q_e_ is reported in mass-based units. Therefore, following Chu [44], in this work, the Temkin model is treated purely as an empirical fit, and only the Temkin binding constant (K_T_) and coefficient of determination R^2^ are reported to represent adsorption affinity and model fitting accuracy.

The Dubinin–Radushkevich (D-R) model [45] was applied in this study primarily to estimate the mean adsorption energy (E), rather than to describe the isotherm fit. The model provides insights into whether the adsorption is physical or chemical in nature. The calculated E values were used to interpret the mechanism but not to predict the isotherm shape. The model (Table 2) assumes that the adsorption occurs in a monolayer of the adsorbent surface, and the adsorption energy varies with the surface site coverage. The mean adsorption energy is represented by the following expression, where β is a constant related to the adsorption energy.

(2) $$E=\frac{1}{\surd 2\beta }$$

**Figure 3:**
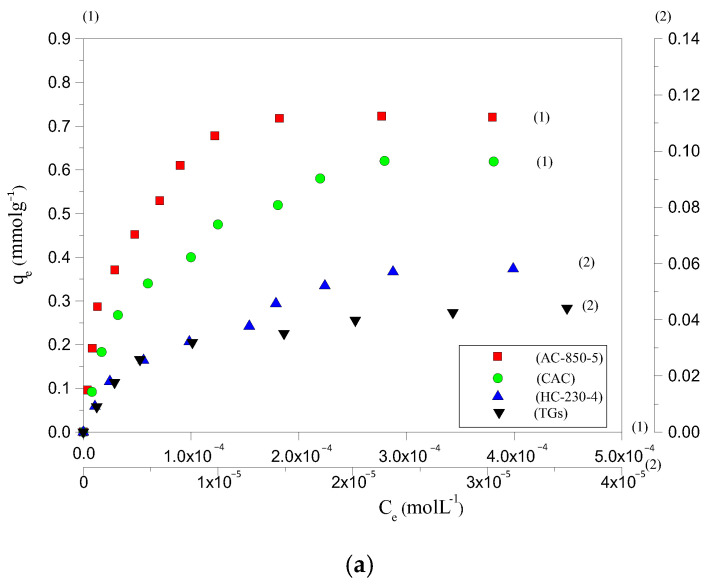

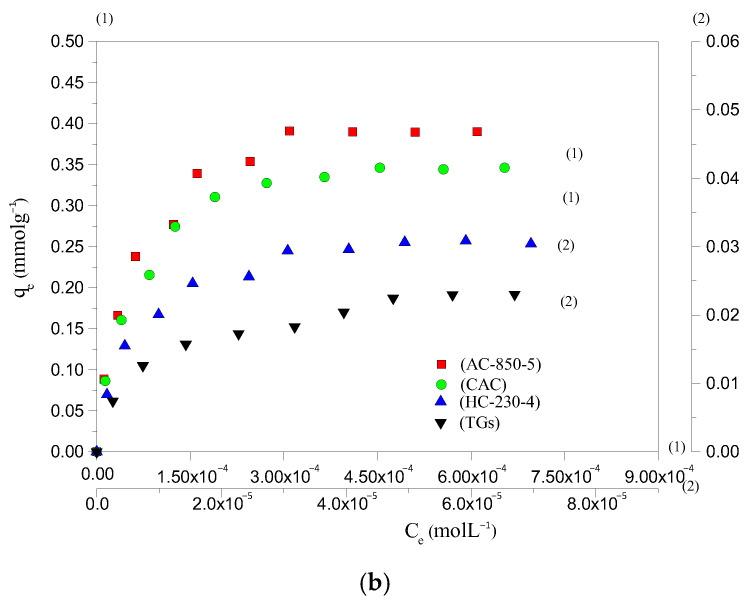
Adsorption isotherm for the removal of (**a**) AYG and (**b**) ABO on four adsorbents, at 25 °C.

The adsorption potential of HC-230-4 and AC-850-5, for AYG and ABO dyes, was determined by plotting equilibrium adsorption, at 25 °C, as a function of equilibrium concentration, as shown in Figure 3a,b, respectively. The parameters obtained based on the application of different equations and models are presented in Table 3.

It can be seen from the isotherms that a maximum amount of 0.058 and 0.030 mmolg^−1^ for AYG and ABO, respectively, were adsorbed by HC-230-4, whereas 0.723 and 0.390 mmolg^−1^ were adsorbed by AC-850-5. While analyzing the isotherm data, it was observed that the prepared hydrochar exhibited very low adsorption capacity for both modeled dyes. However, the activation which allows for the porosity development promotes a significant increase in adsorption capacities of the AC-850-5. The hydrochars are normally prepared at a lower temperature and high pressure than ACs. In this case, the surface area and porosity are less developed in HC-230-4, which is a constraint for the adsorption of large molecules such as the acridine dyes.

The removal efficiency of HC-230-4 and AC-850-5, at 25 °C, for the modeled dyes was also compared with the performance of TGs and with a commercial activated carbon (CAC surface area = 830 m^2^g^−1^, total pore volume = 0.48 cm^3^g^−1^, and average pore size = 2.31 nm). The N_2_ adsorption–desorption isotherm for the CAC was provided in Figure S1. The obtained isotherms are included in Figure 3a,b. It was found that the amount of AYG adsorbed was 0.048, 0.058, 0.637, and 0.723 mmolg^−1^ for TGs, HC-230-4, CAC, and AC-850-5, and the order for the ABO was 0.023, 0.030, 0.346, and 0.390 mmolg^−1^ for TGs, HC-230-4, CAC, and AC-850-5, respectively.

The raw TGs presented the lower adsorption capacity of dyes; this was expected as it has low porosity, where the lignin present reduces the access of the dyes to the pores. The prepared HC-230-4 shows a low adsorption potential, which can be attributed to its smaller surface area (14.41 m^2^g^−1^) and reduced porosity relative to AC-850-5 (729.7 m^2^g^−1^, 0.392 cm^3^g^−1^) and CAC (830 m^2^g^−1^, 0.48 cm^3^g^−1^). Although CAC exhibited a higher surface area and pore volume compared to AC-850-5, its adsorption capacity for AYG and ABO was lower. This difference can be attributed to variations in surface chemistry and pore structure. Since both dyes are cationic, adsorption is not governed solely by electrostatics but also by π–π stacking, hydrophobic interactions, and dye accessibility to the pores. AC-850-5, prepared by the CO_2_ activation of hydrochar at 850 °C, likely retains a greater amount of surface-oxygenated groups (such as hydroxyl and carboxyl functionalities) compared to commercial CAC, which may undergo more extensive high-temperature treatments. These functional groups enhance dye interaction through additional non-electrostatic mechanisms. Furthermore, the slightly smaller average pore size of AC-850-5 (2.15 nm) compared to CAC (2.31 nm) could promote stronger dye confinement effects, increasing van der Waals interactions and stabilizing dye molecules within the pores. These combined factors likely contribute to the higher adsorption capacity observed for AC-850-5. For all adsorbents, a higher adsorption capacity was observed for AYG as compared to ABO, indicating a better affinity between AYG and the adsorbents’ surface.

**Figure 4:**
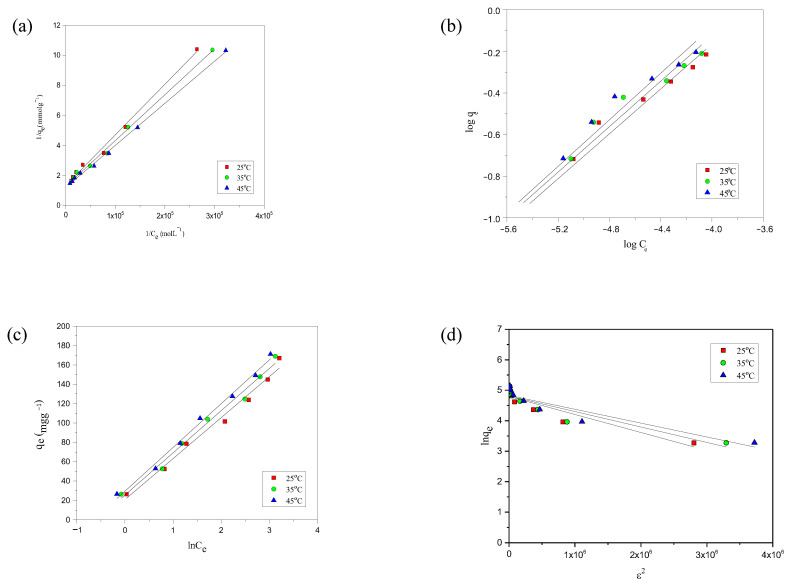
Representation of the (**a**) Langmuir, (**b**) Freundlich, (**c**) Temkin, and (**d**) D-R isotherms for the adsorption of AYG onto AC-850-5 at different temperatures.

**Figure 5:**
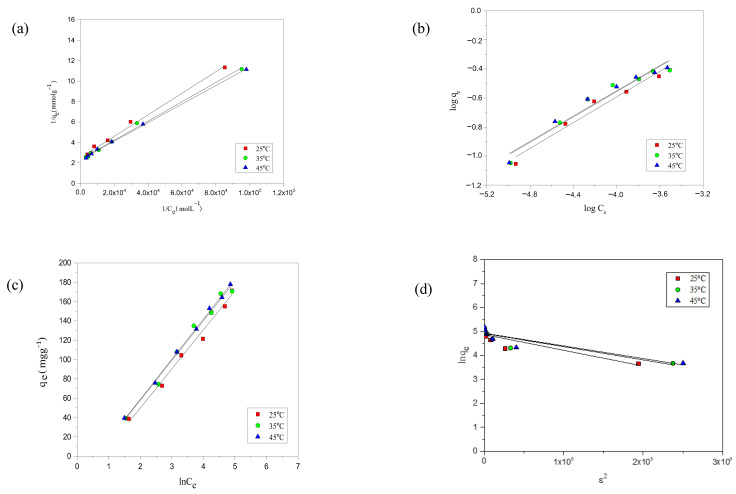
Representation of the (**a**) Langmuir; (**b**) Freundlich; (**c**) Temkin; and (**d**) D-R isotherm for the adsorption of ABO onto AC-850-5 at different temperatures.

The experimental adsorption data for the modeled dyes was evaluated using Langmuir, Freundlich, Temkin, and D-R models. Each model provides a unique perspective on the adsorption mechanism, allowing for a more comprehensive understanding of the interactions between the dyes and the adsorbent’s surfaces. Among these models, the Langmuir isotherm provided the best fit to the experimental data, as evidenced by the high R^2^ values (0.990–0.999) and the close agreement between experimental and calculated adsorption capacities. The D–R model was used solely to estimate the adsorption energy and to confirm the nature of the interaction, not for isotherm fitting. For the Temkin model, only the equilibrium binding constant (K_T_) and the coefficient of determination R^2^ are reported in line with recent recommendations to avoid dimensional inconsistency in mass-based adsorption studies [44]. The extracted model parameters for each isotherm are summarized in Table 3. The analysis of the adsorption data reveals that the Langmuir isotherm fit better to both dyes’ experimental isotherms, as shown by the data presented in Table 3, and supported by the Figure 4a–d and Figure 5a–d for the fit of AYG and ABO onto AC-850-5, obtained at different temperatures.

Similar graphs were obtained for the adsorption of both dyes onto HC-230-4 and are provided in Supplementary Materials (Figures S2 and S3). A notable consensus between experimental adsorption capacities and theoretical Langmuir model estimates emphasizes the model’s effectiveness in describing the observed data. For instance, Acridine Yellow G adsorbed onto HC-230-4 and AC-850-5 exhibited experimental values of 0.058 and 0.723 mmolg^−1^, respectively, which closely matches the Langmuir model’s estimates of 0.059 and 0.756 mmolg^−1^. Similar trends were observed for Acridine Basic Orange 14 (Table 3). Furthermore, the consistently high R^2^ (ranging from 0.990 to 0.999) across all cases confirm the Langmuir model applicability in representing the observed adsorption data. The low adsorption energy values obtained by the Temkin and D-R models, at different temperatures, allows us to confirm that the adsorption of both dyes onto the four adsorbents, as shown in Table 3, is due to the physical adsorption. This observation supports the conclusion that the process is dominated by physisorption, involving weak van der Waals interactions, as reflected by the low E values (all below 8 kJ mol^−1^). It is important to note that, while the D–R model provided insights into adsorption energy, the Langmuir model was superior in describing the adsorption isotherm behavior. El-Shafie and co-workers [46] identified the presence of two stages related to the adsorption of acridine orange onto TTWM500 (thermally treated watermelon rinds at 500 °C). The first stage, which presented an adsorption energy varying from 8.67 kJ/mol to 11.18 kJmol^−1^ was attributed to formation of a first layer, through chemical adsorption. The formation of multilayers, due to the presence of physical adsorption, was identified by the presence of adsorption energy values ranging from 1.90 to 4.08 kJmol^−1^ [46].

### 2.4. Effect of Temperature

**Figure 6:**
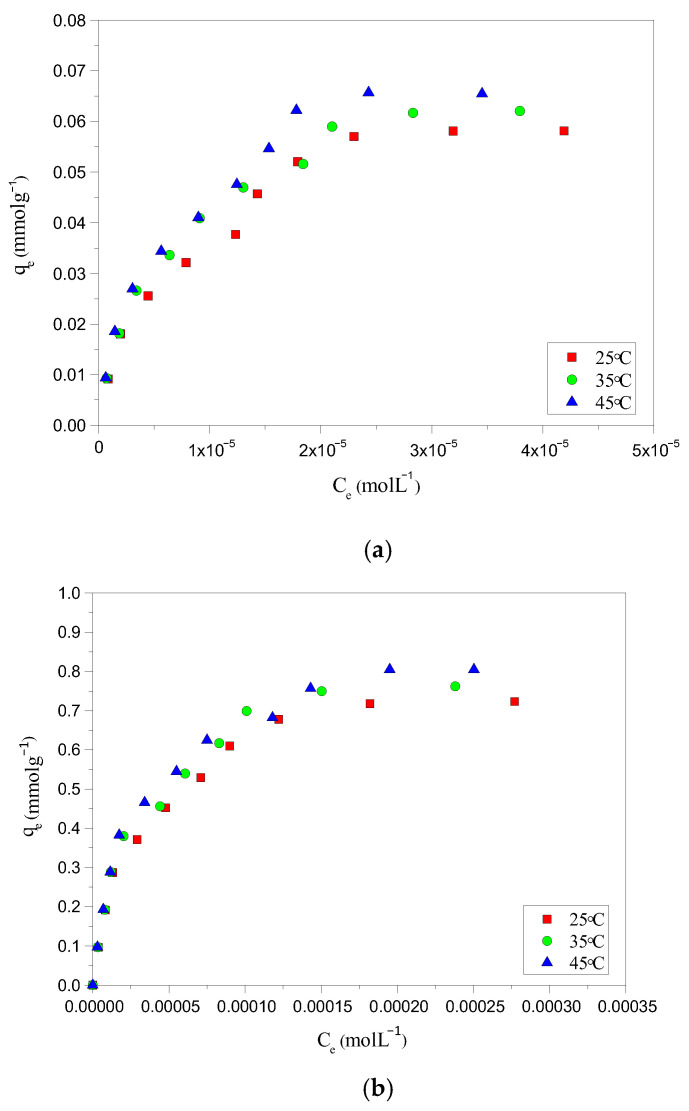
Adsorption isotherm for the removal of AYG onto (**a**) HC-230-4 and (**b**) AC-850-5 at different temperatures.

Temperature plays a vital role in understanding the adsorption process, as it can significantly influence the thermodynamic driving forces, potentially favoring either endothermic or exothermic adsorption mechanisms. The adsorption studies of AYG and ABO over HC-230-4 and AC-850-5 were conducted at temperatures ranging from 25–45 °C. The results depicted in Figure 6a,b for AYG adsorption onto HC-230-4 and AC-850-5, clearly show the influence of temperature on the adsorption process.

Similar graphs were obtained for the adsorption of ABO dye onto HC-230-4 and AC-850-5, and are provided in Supplementary Materials (Figure S4). Figures reveal a positive co-relation between adsorption capacity and temperature. These results suggest that the adsorption process is mainly endothermic in nature, as found previously by Temkin and D-R models. The adsorption of AYG increased from 0.058 to 0.066 mmolg^−1^ and from 0.723 to 0.805 mmolg^−1^, whereas, in the case of ABO, it increased from 0.030 to 0.036 mmolg^−1^ and 0.390 to 0.441 mmolg^−1^ on HC-230-4 and AC-850-5, respectively, when the temperature raised from 25 °C to 45 °C. The increase in the dyes adsorbed was attributed to their diffusion rate in the solution. Increasing the temperature accelerated the diffusion across the external liquid film and inside the pores, which decreased the solution viscosity, and promoted an increase in the adsorption [47,48].

### 2.5. Possible Adsorption Interactions

This section discusses the types of interactions between dyes and adsorbent surfaces (electrostatic forces, π–π stacking, H-bonding), inferred from FTIR and structural analysis. Kinetic mechanisms are discussed separately in Section 2.7. There is no one assumption that can adequately explain the adsorption mechanism of adsorbates onto the adsorbents, as numerous factors, including group functionality, surface area, and porous nature of the adsorbent, as well as the structure and the nature of the adsorbate, influence the adsorption process. The analysis of the obtained adsorption isotherms for both modeled dyes revealed that AYG exhibited higher adsorption compared to ABO, on both adsorbents. From Table 4, it is evident that the molecular size of AYG is smaller compared to ABO. A smaller dye molecule presents a higher diffusivity in water and is more readily attached to available adsorption sites [49].

In a general way, the presence of the –CH_3_ group increases hydrophobicity, favoring adsorption. Despite ABO having a higher number of methyl groups in its structure, AYG exhibited higher adsorption on both adsorbents. This inconsistency may be attributed to the molecular size of ABO, as it covers a higher adsorbent surface area than AYG. In this situation, the presence of methyl groups inhibits other molecules of ABO to approach the adsorbent surface.

**Figure 7:**
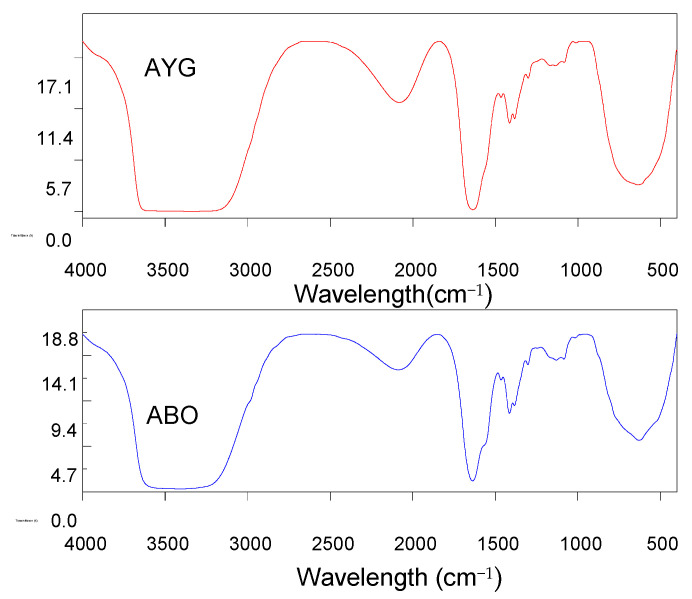
FTIR spectra after the adsorption of AYG and ABO onto AC-850-5.

The dominant interactions in the adsorption of both modeled dyes onto the HC-230-4 surface were electrostatic interactions, H-bonding, and π–π interactions. The FTIR analysis from Table 1 identified the presence of hydroxyl, carboxylic, and phenolic groups on the HC-230-4 surface. The interactions between dye molecules, with a positive charge, and these groups, which bear a negative charge, confirms the dominance of electrostatic interactions in the case of HC-230-4 [13]. The FTIR spectra of AC-850-5 before and after dye adsorption (Figure 7) provide useful insights into the possible interactions between the adsorbent and the acridine dyes. After the adsorption of both AYG and ABO, a slight shift and broadening of the O–H stretching band in the range of 3500–3400 cm^−1^ was observed, which suggests the involvement of hydroxyl groups in hydrogen bonding with the dye molecules. Moreover, the C=O stretching band at 1622 cm^−1^ shifted to 1636 cm^−1^ for AYG and 1637 cm^−1^ for ABO, which indicate the possible interactions between the aromatic rings of the dyes and the carboxyl groups on the adsorbent surface, likely through electrostatic attraction or π–π interactions. Furthermore, the band at 1115 cm^−1^ shifted to 1134 cm^−1^ (AYG) and 1131 cm^−1^ (ABO) after adsorption. This shift is often attributed to π–π stacking interactions between the dye molecules and the aromatic domains of AC-850-5, suggesting a role of non-covalent interactions in dye retention.

The porous structure and surface chemistry of ACs affects the adsorption process to a greater extent [50]. The porosity of AC-850-5 is considered a dominating factor in the adsorption of the modeled dyes, from aqueous solutions. The high surface area (729.70 m^2^g^−1^) and sample porosity (0.392 cm^3^g^−1^) of AC-850-5 allows the dye molecules to adsorb on its surface. The FTIR analyses (Table 1) reveal the presence of carboxylic groups and some aromaticity on the surface of AC-850-5. The electrostatic interactions between both dyes and these groups, along with H-bonding, may enhance the extent of adsorption of the modeled dyes onto AC-850-5.

### 2.6. Thermodynamics

The effectiveness of an adsorption process relies profoundly on its thermodynamic feasibility and underlying behavior. By examining key parameters like Gibb’s free energy (∆G), entropy (∆S°), and enthalpy (∆H°), crucial information about the process’s favorability, randomness, and the nature of the interactions involved can be achieved.

The change in ∆G delivers the information regarding the thermodynamic favorability and spontaneity of the process under the studied conditions. The calculation of the ∆G° for the process is based on the following expression; here, T is the temperature, b and R are the equilibrium constant (from Langmuir) and universal gas constants, respectively.

ΔG = −RT ln b(3)

The change in the ∆H° indicate whether the adsorption process is endothermic or exothermic. Additionally, the change in ∆S° reflects the system’s tendency towards increasing randomness during adsorption. Higher ∆S° values signify increased disorder and suggest a more favorable process. ∆H° and ∆S° can be calculated using the van’t Hoff equation [51], expressed by equation 4, where R is the gas constant:

(4) $$\ln\;b\;=\;-\;\frac{\Delta H{}^{\circ}}{RT}+\;\frac{\Delta S{}^{\circ}}{R}$$

**Figure 8:**
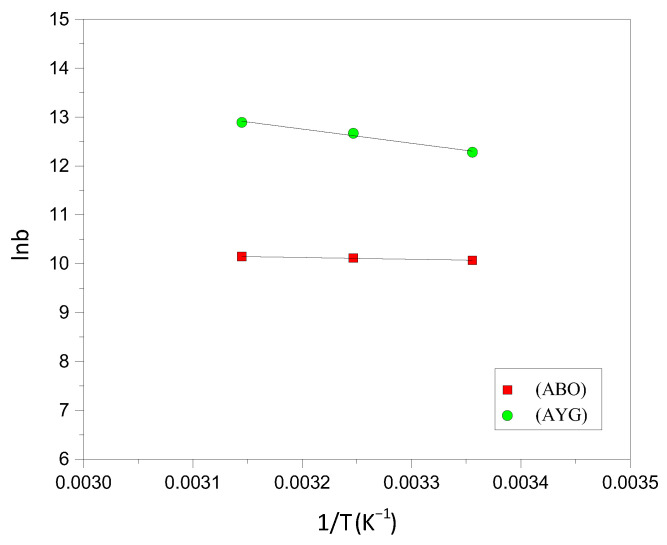
Van’t Hoff plot for the adsorption of AYG and ABO onto AC-850-5.

To investigate the feasibility and nature of the adsorption of AYG and ABO onto HC-230-4 and AC-850-5, experiments were conducted at three distinct temperatures, 25, 35 and 45 °C. Subsequently, the ΔS° and ΔH° of the adsorption process were determined by analyzing the linear relationship between the logarithm of the adsorption capacity (lnb) and reciprocal temperature (1/T). Van’t Hoff plots for the AYG and ABO are shown in Figure 8, for AC-850-5, and the corresponding parameters obtained were reported in Table 5. Similar graphs were obtained for the adsorption of both dyes onto HC-230-4, and are provided in the Supplementary Materials (Figure S5).

For both dyes, the adsorption process was identified as spontaneous and endothermic based on the negative ΔG values obtained at all studied temperatures. Notably, AYG adsorption consistently exhibited more negative ΔG values compared to ABO on both adsorbents, indicating a higher affinity and more favorable adsorption of AYG under the experimental conditions.

### 2.7. Kinetics

Knowledge of the rate at which adsorbates bind to the surface of adsorbents and plays a vital role in understanding and optimizing the processes, mainly with regard to the definition of the residence time [52]. Therefore, to deeply understand the rate controlling mechanism, the kinetic investigations at 25 °C, for the removal of AYG and ABO by HC-230-4 and AC-850-5, were carried out, in a time span of 300 and 240 min, respectively. To understand the mechanisms governing dye adsorption, four well-established kinetic models, viz. pseudo first- and second-order, Elovich and intraparticle diffusion [23,24] were applied.

Pseudo first-order (PFO) rate equation was firstly introduced by Lagergren in 1898 [53]. It describes that the change in the rate of adsorbate uptake by adsorbent is proportional to the difference of equilibrium concentration and the amount of adsorbate adsorbed with time. The equation and the parameters related to the PFO model are given in Table 6.

Pseudo second-order (PSO) rate equation was firstly applied by Ho and Mckay for the modeling of adsorption of lead onto peat in 1966 [54]. Thereafter, the model was widely utilized to validate the kinetic data of the adsorption processes. It describes that the rate-limiting step for the process may be the chemisorption, where forces are involved during the adsorption process in the electron-exchange between the adsorbate and adsorbent. The equation for the PSO model and the respective parameters are provided in Table 6.

The Elovich model [55] is an empirical model used to elucidate the chemisorption of gas onto solid. The Elovich kinetic equation has been considered in order to define the adsorption rate on the heterogenous surface of the adsorbent; however, no detail on the mechanism for the adsorbate–adsorbent interaction was suggested. The model also assumes that the activation energy increases with the increase in the adsorption time. The related equation and the model parameters for the Elovich equation are provided in Table 6.

Weber and Morris in 1962 [56] proposed a model to describe the intraparticle diffusion process, as shown in Table 6. The model describes that the adsorption of an adsorbate, from a solution, to the adsorbent surface involves bulk diffusion, mass transfer of adsorbate (film diffusion), pore diffusion (intraparticle diffusion), and adsorptive attachment. The second and third steps were crucial in deciding the mechanism involved during the adsorption process. The intraparticle diffusion (IPD) model [56] describes the rate limiting step during adsorption and assists in elucidating the exact mechanism for the adsorption process. A plot between q_t_ vs. √t is used to estimate the parameters, k_id_ (rate constant) and C (intercept of ID). According to the model, the intraparticle diffusion controls the process if the obtained plot is a straight line and passes through the origin. However, deviation from the straight line or non-zero intercept suggests that the adsorption is controlled by different processes (bulk diffusion, mass transfer of adsorbate, and adsorptive attachment).

**Figure 9:**
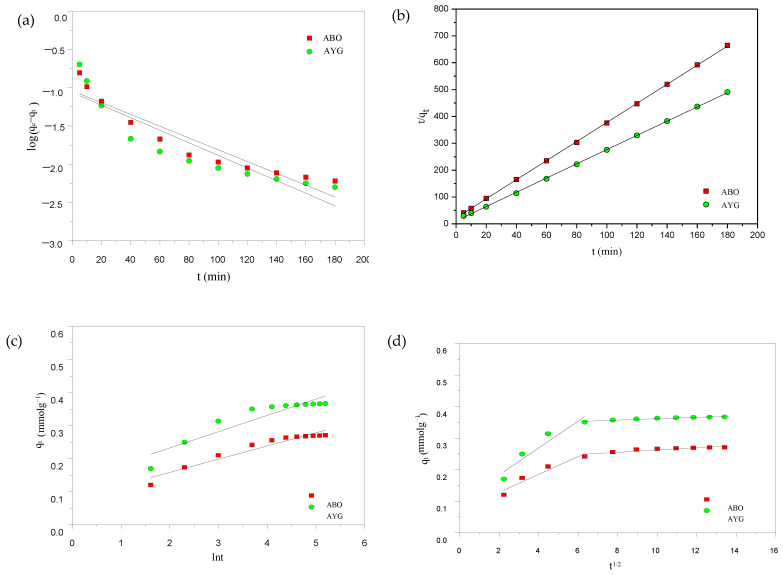
(**a**) PFO; (**b**) PSO; (**c**) Elovich; and (**d**) intraparticle kinetic plots for the adsorption of AYG and ABO onto AC-850-5 (C_i_: 4 × 10^−4^ moldm^−3^; T: 25 °C).

The PFO, PSO, and Elovich models for AYG and ABO onto AC-850-5 are shown in Figure 9a–d and the obtained parameters are presented in Table 7. The graphs were also obtained for the adsorption of both dyes onto HC-230-4, and are provided in Supplementary Materials (Figure S6). On applying the pseudo first-order rate equation to the experimental data in Figure 9a, the theoretical adsorption capacity did not agree well with the experimental values. Moreover, the low value of the regression coefficient indicated that the pseudo first-order model did not satisfactorily fit the experimental data in Table 7. In contrast, applying the pseudo second-order equation yielded adsorption capacity values q_e_ (theoretical) closely matching to those obtained experimentally, as shown in Figure 9b and data presented in Table 7. Additionally, the R^2^ obtained for the equation was in close to unity, indicating a good fit for the equation. These results suggest that the adsorption kinetics follow a pseudo-second-order behavior, indicating that the rate-limiting step may be related to surface site interactions and adsorption capacity availability [57]. 

Furthermore, to better compare the initial adsorption kinetics between the adsorbents and adsorbates, the initial adsorption rate (h) was calculated from the pseudo-second-order model using the equation h = k_2_$q_{e}^{2}$. The values obtained were 0.00871 mmol g^−1^ min^−1^ for AYG and 0.00463 mmol g^−1^ min^−1^ for ABO on HC-230-4, and 0.089 mmol g^−1^ min^−1^ for AYG and 0.0461 mmol g^−1^ min^−1^ for ABO on AC-850-5. These results highlight that the AC-850-5 exhibits a significantly faster initial adsorption rate, owing to its higher surface area and porosity. Additionally, the higher h values for AYG in both cases indicate a better initial interaction and affinity compared to ABO.

The Elovich model was also applied and the corresponding plot and parameters for the dyes onto AC-850-5 are shown in Figure 9c and the parameters are presented in Table 7. The low regression coefficient (0.862–0.955) obtained for this model indicates its inapplicability. Therefore, among these three models, pseudo second-order model equation was identified as the most appropriate for describing the adsorption kinetics of AYG and ABO on HC-230-4 and AC-850-5.

The intraparticle diffusion model (IPD) was further tested to elucidate the mechanism and rate governing step for the adsorption of AYG and ABO dye onto AC-850-5. The obtained plots for the modeled dyes are given in Figure 9d and the corresponding parameters are shown in Table 7. In Figure 9d, two straight lines were identified and no one passed through the origin, which is consistent with a complex adsorption process [58].

The intraparticle diffusion rate constant K_id_ (K_id1_ for the first stage and K_id2_ for the second stage) and the value of C (C_1_ for the first stage and C_2_ for the second stage) were obtained from the plot of q_t_ versus t^1/2^. From the Table 7, it can be observed that the value of K_id2_ is lower than K_id1_ for both dyes, which shows that intraparticle diffusion is the rate limiting step. Therefore, it can be concluded that the adsorption of AYG and ABO onto AC-850-5 follows a complex mechanism, where the four identified mechanisms can play an important role.

## 3. Materials and Methods

### 3.1. Precursor, Adsorbents, Adsorbates, and Chemicals

*Tectona grandis* seeds (TGs) utilized in this work were obtained locally. The as-obtained TGs were thoroughly washed to remove the adhered dust and impurities, and then dried. The dried seeds were then ground and sieved to obtain a homogeneous size (10–30 mesh) and further dried in an oven, at 105 °C, for 24 h. The prepared sample was kept in air tight containers for further use. The adsorbates, Acridine basic orange 14 was supplied by TCI chemicals, Tokyo, Japan, and Acridine yellow G was purchased from Loba Chemie, Mumbai, India. The properties of both dyes are included in Table 4. NaOH and HCl were purchased from Rankem, Gurugram, India, and Merck, Darmstadt, Germany, respectively.

### 3.2. Adsorbent’s Production and Characterisation

This work is the continuation of our previously published work. Production conditions and some of the key characterization findings (TGA, FE-SEM, FTIR, XRD, textural properties, and surface chemistry) of the prepared adsorbents, HC-230-4, AC-850-5, and raw *Tectona grandis* seeds, adapted from the previously published article, [34], are presented in the Table 1. The commercial activated carbon used in this study, for comparative purposes, was procured from Merck, Germany.

### 3.3. Adsorption Studies

The adsorption studies were performed employing the batch experiments. The effect of different parameters such as pH (2–12), contact time (0 to 300 min), the initial dye concentration (5 × 10^−5^, 6 × 10^−5^ moldm^−3^ for HC-230-4, and 4 × 10^−4^, 5 × 10^−4^ moldm^−3^, for AC-850-5) and the temperature (25, 35 and 45 °C) were evaluated. The experiments were carried out in a temperature-controlled shaking assembly (JSGW, India), having a stirring speed of 40–140 cycles per minute. The pH values of the dye solution were adjusted by using HCl and NaOH solutions (0.1 mol dm^−3^) and a pH meter (HI2210, Hanna, instruments, Smithfield, USA). After the desired contact time, the suspensions containing the adsorbents and dyes were filtered and the concentration of the remaining dyes was evaluated, at their respective λ_max,_ by a UV–Vis spectrophotometer (UV-1800, Shimadzu, Kyoto, Japan). Each experiment was repeated thrice to minimize the experimental errors.

## 4. Conclusions

The seeds of the *Tectona grandis* have been successfully utilized as a starting material to prepare adsorbents (hydrochar and activated carbon) capable of adsorbing cationic acridine dyes from aqueous phase. The results demonstrated that the developed HC-230-4 but mainly the AC-850-5 exhibited a significantly higher surface affinity for AYG and ABO dyes as compared to raw *TGs*. The AC-850-5 presents a well-developed porous structure which reflects on the amount of acridine dyes adsorbed. The maximum adsorption capacity found was 15.9 and 198 mgg^−1^ for acridine yellow G onto hydrochar and activated carbon, respectively, while it was found to be 13.5 and 171 mgg^−1^ for acridine basic orange 14. The maximum adsorption occurs under the conditions of the pH ranging between 5 and 9, for both adsorbents, and a contact time of 240 and 180 min for HC-230-4 and AC-850-5, respectively. It was found that the adsorption was an endothermic process, on both adsorbents (positive values of ΔH°). The values of ΔG° corroborate the spontaneity and the physical nature of the adsorption process. Furthermore, the adsorption studies carried out in this work showed that the adsorption of both dyes on both adsorbents followed the Langmuir isotherm models. The kinetic study confirmed that the adsorption of both dyes onto HC-230-4 and AC-850-5 follows a pseudo-second-order model, with intraparticle diffusion acting as a rate-limiting step, suggesting a multi-step adsorption mechanism.

## Figures and Tables

**Table 1:** Characteristics of adsorbents (*Tectona grandis* seeds, HC-230-4 and AC-850-5 [34].

Ultimate analysis—The carbon content for the *Tectona grandis* seeds was 48.47% which, upon hydrothermal carbonization, increased up to 66.19%. The AC presents a carbon content reaching 83.57%, indicating that hydrochar is a good precursor for AC production.
Thermogravimetric analysis (TGA)—The TGA curves for *Tectona grandis *seeds and hydrochar showed a three-stage weight loss trend upon analysis. Firstly, near 100 °C that might be associated to the loss of moisture content. A major weight loss was verified between 200 and 400 °C, viz. 31.8% for TGs and 27% for HC-230-4, which was associated with the hemicellulose and cellulose decomposition. The last weight loss of 12.2 and 12.9%, attributed to the oxidation of char, was observed in TGs and HC-230-4, near 500 °C. For the AC-850-5, a third stage starts after 500 °C and was attributed to lignin degradation (175–800 °C) and to the development of new carbon structures.
Morphological examination (FE-SEM)—TGs showed a smooth surface morphology, but after hydrothermal carbonization, it showed the development of spherical shapes on the hydrochar’s surface. The developed spherical shapes were present even after physical activation, with more extensive agglomerated microspheres.
Functional group examination (FTIR)—Bands at 3405, 3379, and 3400 cm^−1^ were observed in TGs, HC-230-4, and AC-850-5, respectively, due to the presence of hydroxyl groups. In the three adsorbents, bands in the region near 1600 cm^−1^ were observed and attributed to the presence of carboxylic groups.
Structural examination (XRD)—Two peaks at 2θ 15^o^ and 22^o^ were observed in TGs and HC-230-4, which are the characteristic peaks of cellulose. The activation process promoted a broadening of both peaks, showing the degradation of cellulosic structure and the formation of an amorphous material. The peak at 2θ 40^o^ confirmed the presence of a typical activated carbon, with more ordered graphitic structures.
Textural analysis (nitrogen adsorption at 77 K)—The apparent surface area and total pore volume, of the developed activated carbon (AC-850-5), obtained by BET method, was 729.7 m^2^g^−1^, 0.392 cm^3^g^−1^. The micro-pore volume and mean pore size, obtained by BJH method, were 0.286 cm^3^g^−1^ and 2.15 nm, respectively. The TGs and HC-230-4 were also subjected to BET analysis and a low surface area was obtained, respectively, 3.6 and 14.41 m^2^g^−1^.
Surface chemistry (Boehm method and pHpzc)—Total acidic groups, on HC-230-4 and AC-850-5, were found to be 1.32 and 0.432 mmolg^−1^, respectively, whereas the concentration of total basic groups were found to be 0.19 mmolg^−1^ on HC-230-4 and 0.21 mmolg^−1^ on AC-850-5, using Boehm’s titration method. The pH at the zero point of charge (pH_pzc_) was calculated by the mass titration method [35] and was found to be 4.3 and 6.2 for HC-230-4 and AC-850-5, respectively.

**Table 2:** Mathematical expressions and related parameters for Langmuir, Freundlich, Temkin, and D-R isotherm models.

Type of Isotherm	Equation	Plotted Between	Parameters
Langmuir	1/q_e_ = 1/q_max_ + 1/q_max_bC_e_	1/q_e_ vs. 1/C_e_	b (Lmol^−1^); Langmuir adsorption coefficient
Freundlich	log q_e_ = logK_f_ + (1/n) log C_e_	logq_e_ vs. logC_e_	K_f_ (mmolg^−1^.mol^−1/n^⋅L^1/n^); isotherm constant related to the adsorption capacity, *n*; heterogeneity factor constant
Temkin	q_e_ = B_T_ ln K_T_ + B_T_ lnC_e_	q_e_ vs. lnC_e_	K_T_ (Lmg^−1^)_:_ Temkin equilibrium binding constant (empirical)
D-R	ln q_e_ = lnq_m_ − β_DR_ε^2^ ε = RT ln(1 + 1/C_e_)	ln q_e_ vs. ε^2^	q_m_ (mgg^−1^); D-R monolayer capacity; β_DR_ (mol^2^kJ^−2^); Constant related to the adsorption energy; ε; Polanyi potential

In Table 2, C_e_—the adsorbate concentration at equilibrium (mg L^−1^); q_e_—the amount adsorbed at C_e_ (mmol g^−1^), and q_max_—Langmuir monolayer capacity (mmol g^−1^).

**Table 3:** Adsorption isotherm parameters for the adsorption of Acridine Yellow G (AYG) and Acridine Basic Orange 14 (ABO) dye onto hydrochar (HC-230-4) and derived activated carbon (AC-850-5) at different temperatures.

Adsorbent/Temperature	Dyes	Experimental		Langmuir				Freundlich			Temkin			D-R	
		q_exp_	q_exp_	q_max_	q_max_	B	R^2^	K_f_	N	R^2^	K_T_	R^2^	q_m_	ε	R^2^
		mgg^−1^	mmolg^−1^	mmolg^−1^	mgg^−1^	Lmol^−1^		mmol⋅g^−1^⋅mol^−1/n^⋅L^1/n^			Lmg^−1^		mgg^−1^	kJmol^−1^	
HC-230-4/25 °C	AYG	15.9	0.058	0.054	14.9	2.35 × 10^5^	0.992	16.7	1.89	0.979	7.14	0.944	10.9	2.31	0.899
	ABO	13.5	0.030	0.031	13.7	2.20 × 10^5^	0.995	1.63	2.58	0.960	4.88	0.988	11.1	1.42	0.889
HC-230-4/35 °C	AYG	16.9	0.062	0.057	15.6	2.66 × 10^5^	0.997	17.8	1.90	0.980	8.50	0.987	11.4	2.47	0.912
	ABO	14.8	0.033	0.032	14.2	2.33 × 10^5^	0.995	1.70	2.59	0.957	5.28	0.988	11.5	1.48	0.891
HC-230-4/45 °C	AYG	18.0	0.066	0.061	16.3	2.88 × 10^5^	0.996	18.0	1.92	0.977	9.76	0.978	12.0	2.60	0.913
	ABO	15.7	0.036	0.035	15.5	2.48 × 10^5^	0.994	2.17	2.52	0.957	5.55	0.988	12.5	1.59	0.894
AC-850-5/25 °C	AYG	198	0.723	0.756	207	3.92 × 10^4^	0.995	97.7	1.86	0.958	1.63	0.984	121	0.92	0.862
	ABO	171	0.390	0.402	176.2	2.36 × 10^4^	0.994	15.1	2.26	0.969	0.46	0.985	131	0.28	0.802
AC-850-5/35 °C	AYG	209	0.762	0.794	217	4.13 × 10^4^	0.996	114	1.83	0.948	1.80	0.986	123	0.99	0.852
	ABO	182	0.416	0.430	188	2.47 × 10^4^	0.996	15.4	2.29	0.948	0.55	0.987	137	0.30	0.815
AC-850-5/45 °C	AYG	220	0.805	0.838	229	4.26 × 10^4^	0.998	142	1.79	0.949	1.92	0.992	125	1.05	0.863
	ABO	193	0.441	0.433	190	2.55 × 10^4^	0.999	15.5	2.30	0.961	0.56	0.997	138	0.31	0.826

**Table 4:** Molecular structures and properties of Acridine Yellow G (AYG) and Acridine Basic Orange 14 (ABO) dyes.

Acridine Yellow G (AYG)	Properties
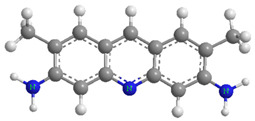 .HCl	CAS No.-135-49-9 and C.I. No.-46025 Dye Class—Acridine Molecular Formula—C_15_H_15_N_3_.HCl Topological polar surface area—64.9 Å^2^ Molecular Weight—273.76 g mol^−1^ pH (stock solution)—8.3 λ_max_—445 nm Water solubility—1 mg mL^−1^, at 20 °C
**Acridine Basic Orange 14 (ABO)**	
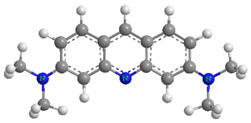 .1/2 ZnCl2.HCl	CAS No.-10127-02-03 and C.I. No.-46005 Dye class—Acridine Molecular formula—C_17_H_19_N_3_.0.5ZnCl_2_.HCl Topological polar surface area—19.4 Å^2^ Molecular weight—369.96 g mol^−1^ pH (stock solution)—6.4 λ_max_—494 nm Water solubility—6 mg mL^−1^, at 20 °C

**Table 5:** Thermodynamic parameters for the adsorption of Acridine Yellow G (AYG) and Acridine Basic Orange 14 (ABO) onto HC-230-4 and derived AC-850-5.

Adsorbent	Adsorbate	ΔG (kJ mol^−1^)			ΔH° (J mol^−1^)	ΔS° (J mol^−1^ K^−1^)
		25 °C	35 °C	45 °C		
HC-230-4	AYG	−30.6	−31.9	−33.2	8.06 × 10^3^	129.9
	ABO	−30.4	−31.6	−32.8	4.74 × 10^3^	118.2
AC-850-5	AYG	−26.7	−27.7	−28.7	3.28 × 10^3^	98.9
	ABO	−24.9	−25.9	−26.8	3.03 × 10^3^	93.9

**Table 6:** Mathematical expressions and related parameters for pseudo-first order, pseudo-second order, Elovich, and intraparticle diffusion models (q_e_ (mmolg^−1^)—equilibrium capacity; q_t_ (mmolg^−1^); adsorption capacity at time t).

Kinetic Model	Equations	Plotted Between	Parameters
Pseudo 1st order	$log(qₑ-qₜ)=logqₑ-\frac{k_{1}}{2.303}t$	log (q_e_ − q_t_ ) vs. T	k_1_; 1st-order rate constant of adsorption
Pseudo 2nd order	$\frac{t}{q_{t}}=\frac{1}{k_{2}q_{e}^{2}}+\frac{1}{q_{e}}$ t	$\frac{t}{q_{t}}\;$vs. t	k_2_; 2nd-order rate constant of adsorption
Elovich	q_t_ = 1/β ln(αβ) + 1/β ln(t)	q_t_ vs. ln(t)	β (gmmol^−1^); desorption rate constant α (mmolg^−1^·min^−1^); Initial adsorption rate constant
Intraparticle diffusion	q_t_ = K_id_. √t + C	q_t_ vs. √t	K_id_; Rate constant of IDC; intercept of ID

**Table 7:** Kinetic parameters for the adsorption of Acridine Yellow G (AYG) and Acridine Basic Orange 14 (ABO) dye onto hydrochar HC-230-4 and derived activated carbon (AC-850-5).

Kinetic Model	Parameters	HC-230-4		AC-850-5	
		AYG	ABO	AYG	ABO
		6 × 10^−5^ M	6 × 10^−5^ M	4 × 10^−4^ M	4 × 10^−4^ M
Experimental	q_e_ (mmolg^−1^)	0.046	0.029	0.372	0.277
Pseudo First Order	q_e_(cal) (mmolg^−1^)	0.010	0.008	0.073	0.072
	K_1_ (min^−1^)	0.015	0.014	0.013	0.013
	R^2^	0.856	0.891	0.826	0.858
Pseudo Second Order	q_e_ (cal) (mmolg^−1^)	0.046	0.029	0.374	0.279
	K_2_ (gmmol^−1^min^−1^)	4.12	5.51	0.643	0.603
	R^2^	0.999	0.999	0.999	0.999
Elovich	α (mmolg^−1^min^−1^)	0.112	0.039	1.92	0.442
	β (gmmol^−1^)	177.78	251.38	24.1	27.7
	R^2^	0.908	0.955	0.862	0.927
Intraparticle Diffusion	K_id1_	-	-	0.039	0.028
	C_1_	-	-	0.119	0.072
	R^2^	-	-	0.921	0.973
	K_id2_	-	-	0.002	0.003
	C_2_	-	-	0.335	0.234
	R^2^	-	-	0.873	0.775

## Data Availability

Data will be made available upon request.

## Supplementary Materials

The following supporting information can be downloaded at https://www.mdpi.com/article/10.3390/molecules30193989/s1. Figure S1. N_2_ adsorption desorption isotherm of commercial activated carbon. Figure S2. (a) Langmuir (b) Freundlich, (c) Temkin and (d) D-R isotherm for the adsorption of AYG onto HC-230-4 at different temperatures. Figure S3. (a) Langmuir (b) Freundlich, (c) Temkin and (d) D-R isotherm for the adsorption of ABO onto HC-230-4 at different temperatures. Figure S4. Adsorption isotherm for the removal of ABO onto (a) HC-230-4 and (b) AC-850-5 at different temperatures. Figure S5. Van’t Hoff plot for the adsorption of AYG and ABO onto HC-230-4 at 25 °C. Figure S6. (a) PFO; (b) PSO and (c) Elovich kinetic plots for the adsorption of AYG and ABO onto HC-230-4 (Ci: 6 × 10^−5^M; T: 25 °C).
