# Supplementary material for: Assessment of Hydrochar and Porous Carbon from Tectona Grandis Seeds for Removal of Acridine Dyes
Source: Molecules. 2025 Oct 4;30(19):3989. doi: 10.3390/molecules30193989 (PMC12526478; doi:10.3390/molecules30193989)
Supplement: Supplementary file 1 [file molecules-30-03989-s001.zip › molecules-3715300-supplementary.pdf]

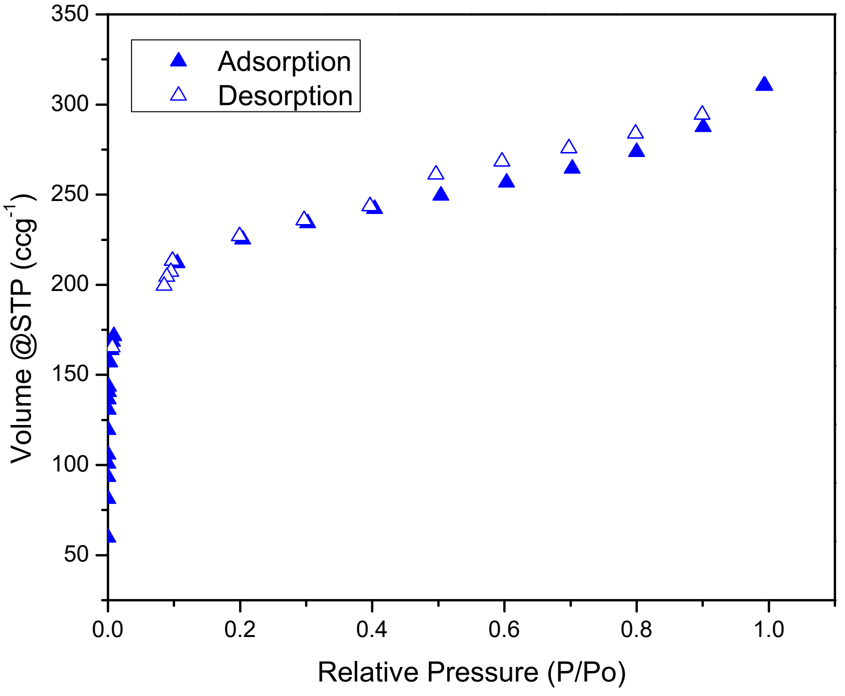

Figure S1. N_2 adsorption desorption isotherm of commercial activated carbon.

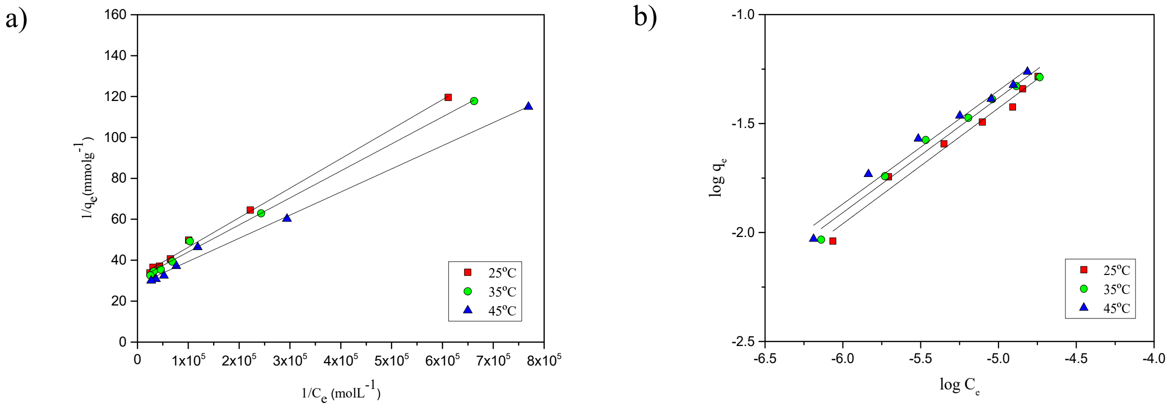

c)

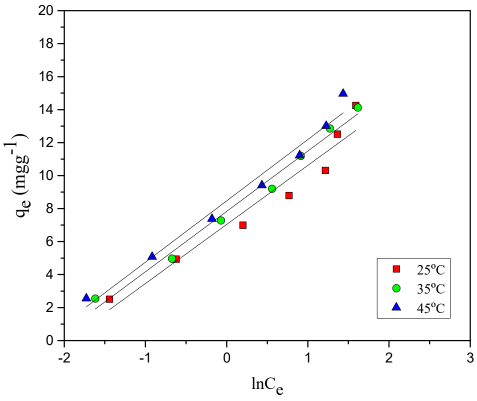

d)

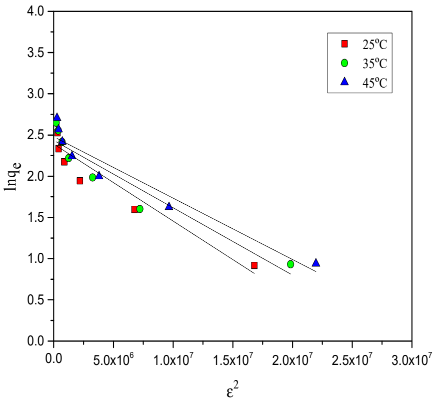

Figure S2 a) Langmuir b) Freundlich, c) Temkin and d) D-R isotherm for the adsorption of AYG onto HC-230-4 at different temperatures.

a)

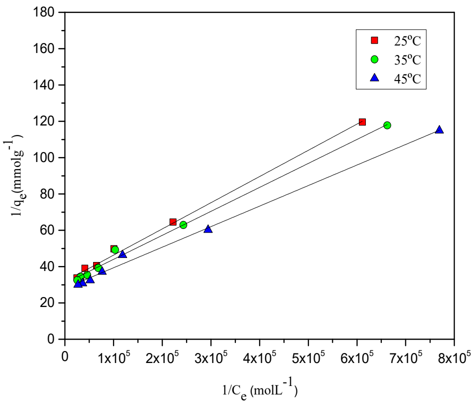

b)

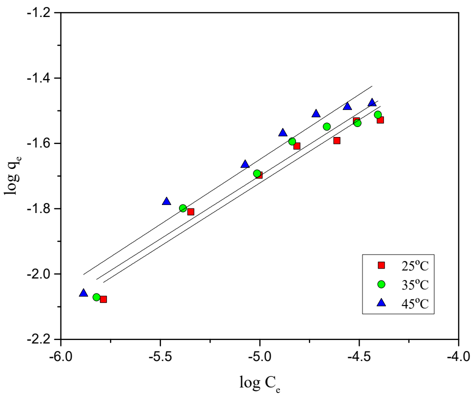

c)

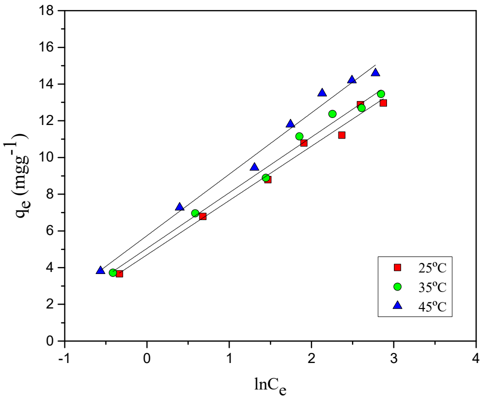

d)

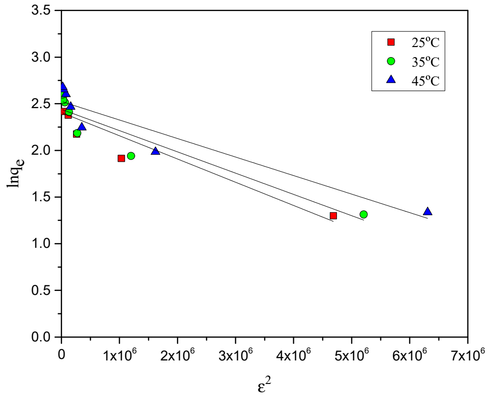

Figure S3 a) Langmuir b) Freundlich, c) Temkin and d) D-R isotherm for the adsorption of ABO onto HC-230-4 at different temperatures.

a) b)

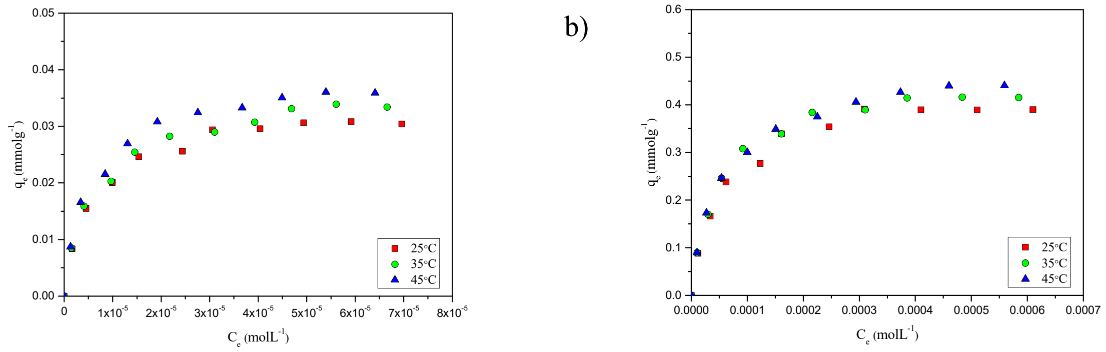

Figure S4. Adsorption isotherm for the removal of ABO onto a) HC-230-4 and b) AC-850-5 at different temperatures

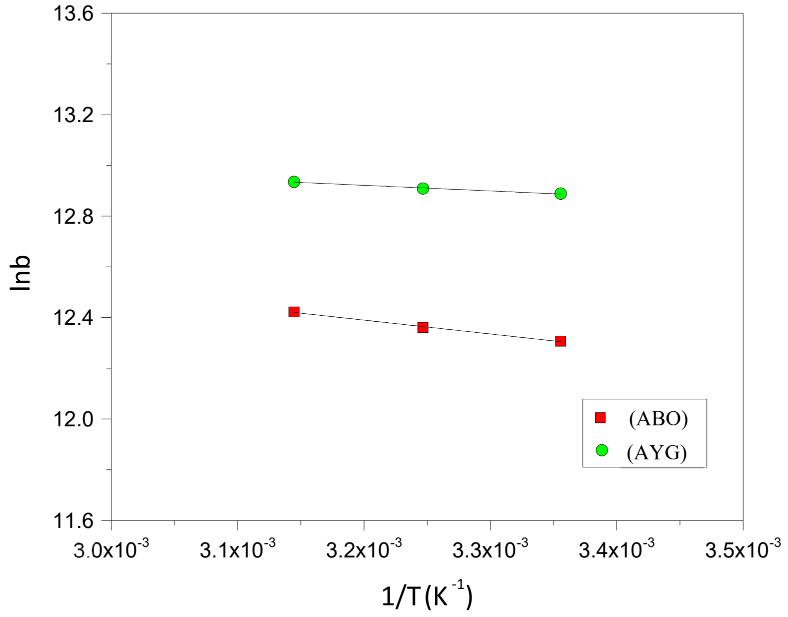

Figure S5 Van't Hoff plot for the adsorption of AYG and ABO onto HC-230-4 at 25°C

a)

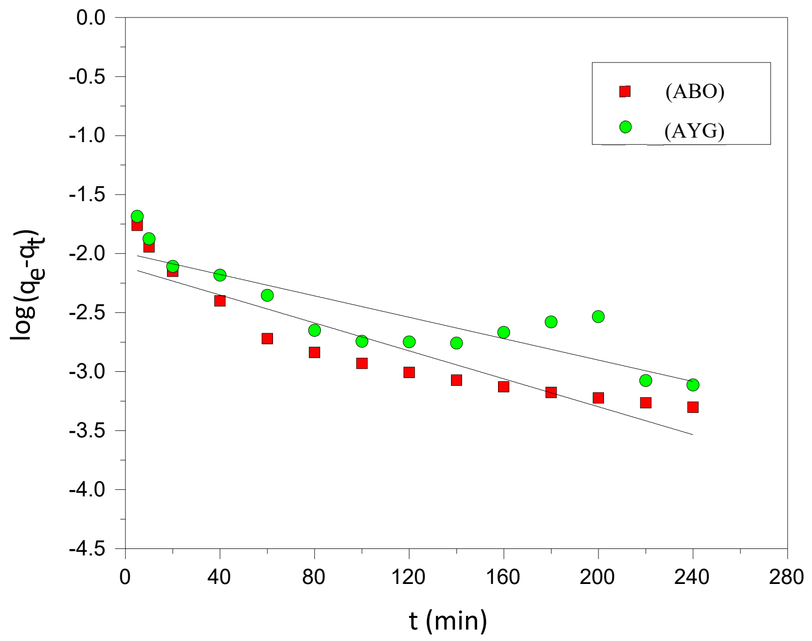
0.0
-0.5
-1.0
-1.5
-2.0
-2.5
-3.0
-3.5
-4.0
-4.5
log (qe-qt)
0
40
80
120
160
200
240
280
t (min)
(ABO)
(AYG)

b)

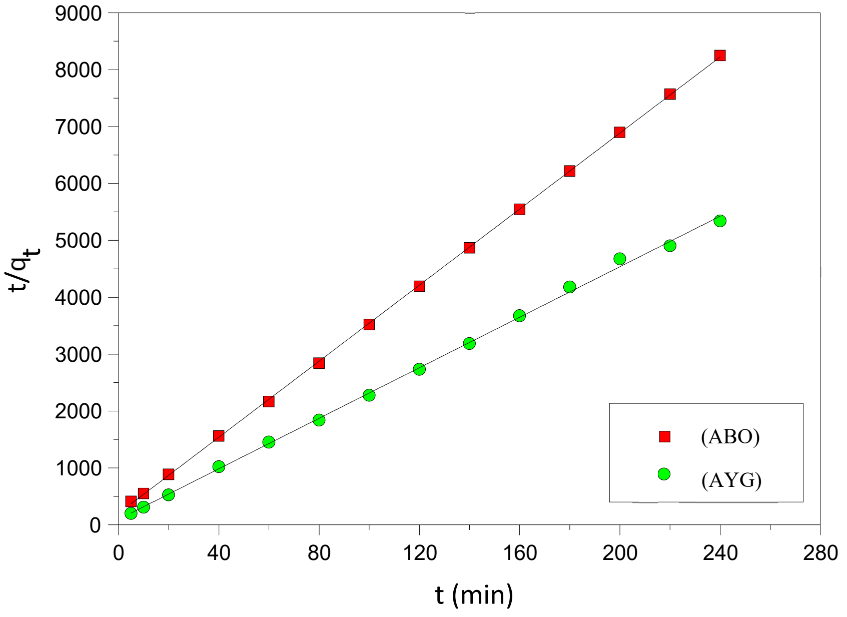
9000
8000
7000
6000
5000
4000
3000
2000
1000
0
t/qt
0
40
80
120
160
200
240
280
t (min)
(ABO)
(AYG)

c)

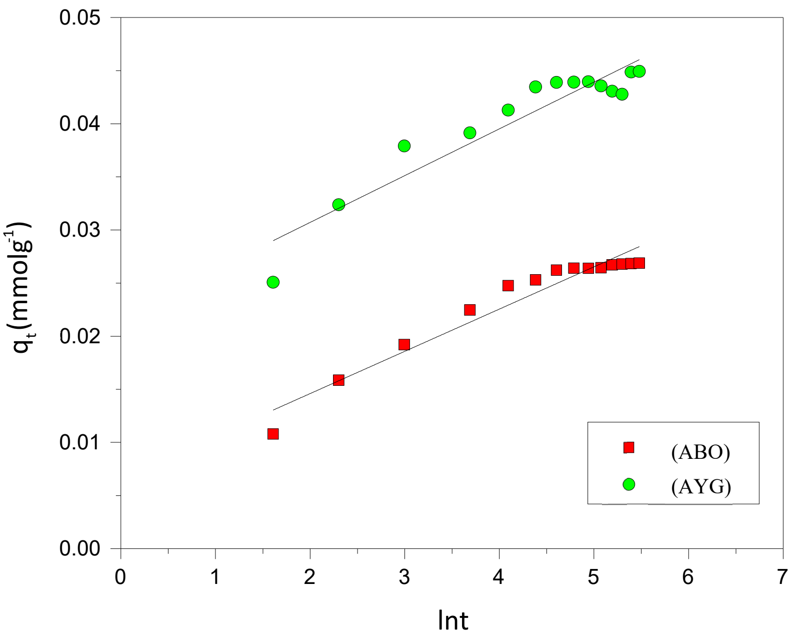

Figure S6 a) PFO; b) PSO and c) Elovich kinetic plots for the adsorption of AYG and ABO onto HC-230-4 (C_i: 6×10^{-5}M; T: 25°C)
